# Unusual upper back presentation of primary cutaneous diffuse large B-cell lymphoma, leg-type with complete regression

**DOI:** 10.1016/j.jdcr.2026.01.019

**Published:** 2026-01-23

**Authors:** Ekaterina Korytnikova, Albert Zhou, Donna Aiudi, Michael Murphy, Philip Kerr

**Affiliations:** Department of Dermatology, UConn Health, Farmington, Connecticut

**Keywords:** B-cell lymphoma, leg type, non-leg location, PET/CT, primary cutaneous diffuse large B-cell lymphoma, regression

## Introduction

Primary cutaneous diffuse large B-cell lymphoma, leg-type (PCDLBCL-LT) is a rare but aggressive subtype of primary cutaneous B-cell lymphoma.^1^^,^^2^ It typically affects elderly women, with a median age of onset around 76 years, and carries a poor prognosis with reported 5-year survival rates near 50%.^1^^,^^2^ Classically, it presents as red-to-purple nodules on the lower extremities although 10% to 15% present at other cutaneous sites.^1^, ^2^, ^3^, ^4^ In the current World Health Organization European Organization for Research and Treatment of Cancer classification, ‘leg-type’ refers to an activated non–germinal center B-cell immunophenotype characterized by BCL-2 and multiple myeloma oncogene 1 co-expression (often with Forkhead box protein P1, IgM, myelocytomatosis positivity, and CD10 negativity), rather than strict anatomic localization to the lower extremities.^1^^,^^2^

Given the aggressive nature, multimodal therapy is often preferred. However, in patients with a solitary lesion, localized disease, or tumors arising at non-leg sites, factors associated with more favorable prognosis, treatment de-escalation to local radiotherapy (RT) may be reasonable, particularly as 2-year overall survival appears comparable to multiagent regimens.^1^^,^^2^ Self-resolution of PCDLBCL-LT without systemic therapy is exceedingly rare, with only a few cases described in the literature.^5^^,^^6^ Here, we report an unusual case of PCDLBCL-LT presenting on the upper back in an elderly male, showing complete clinical and positron emission tomography/computed tomography (PET/CT) resolution after biopsy in the absence of systemic therapy.

## Case presentation

**Fig 1:**
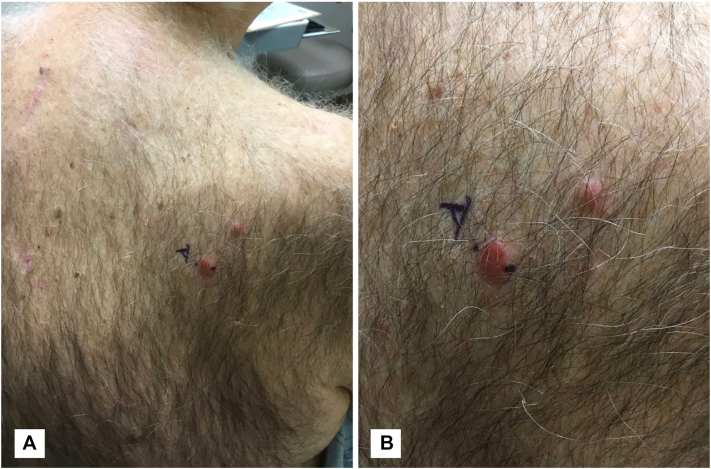
Photographs (**A,B**) of the upper back at initial presentation showing 2 erythematous solitary plaques.

A 79-year-old man presented with a new pruritic lesion on the right upper back. Examination revealed 2 well-demarcated, erythematous, firm, discrete flat-topped thin plaques (Fig 1). He denied fevers, night sweats, and weight loss, and physical examination showed no lymphadenopathy or splenomegaly. Laboratory studies revealed normal blood counts, uric acid, and lactate dehydrogenase.

**Fig 2:**
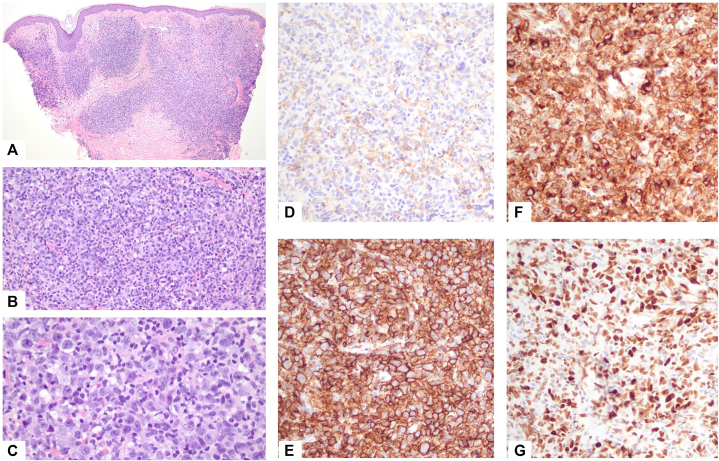
Histopathology of a punch biopsy of the demonstrating multinodular infiltrate of enlarged mononuclear cells, hematoxylin-eosin; magnifications **(A)** ×40; **(B)** ×100; **(C)** ×400. Negative staining for CD10 **(D)**, positive staining for CD20 **(E)**, BCL-2 **(F)**, BCL-6 **(G)**.

A shave biopsy was obtained from the inferior of the 2 plaques; the second was not sampled. There was a brisk, tumor-infiltrating multinodular infiltrate extending from the superficial dermis to the upper subcutis consisting of enlarged mononuclear cells with irregular nuclear contours, fine chromatin, small nucleoli, and scant cytoplasm, admixed with small lymphocytes (Fig 2), without involvement of the overlying epidermis. Immunohistochemistry showed positivity for leukocyte common antigen (CD45), CD20, B-cell lymphoma (BCL)-2, BCL-6, multiple myeloma oncogene 1, Forkhead box protein P1, IgM, and myelocytomatosis; and negative for IgD, CD5, CD10, CD23, CD30, CD34, CD138, cyclin D1, AE1/AE3, and Sry-box transcription factor 10. Ki-67 revealed high proliferative rate; CD3 and CD5 highlighted small background T-cells. The case was reviewed in consultation with the hematopathology laboratory at the National Institutes of Health/National Cancer Institute, with a consensus diagnosis most consistent with diffuse large B-cell lymphoma leg-type with non-germinal center B-cell phenotype by Hans algorithm. The patient was referred to oncology for further management and evaluation.

**Fig 3:**
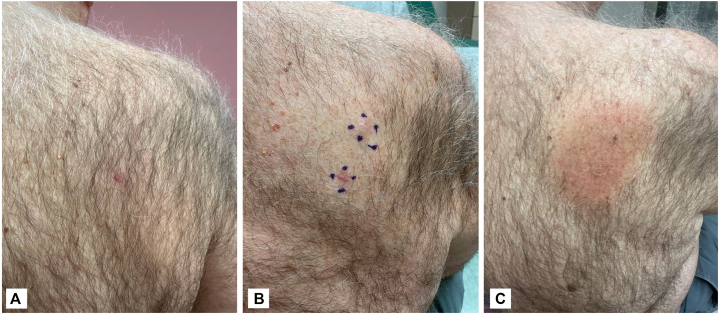
Photography of the upper back at follow-up showing resolution of lesions. **A,** At 6 weeks follow up before RT. **B,** At 3 months follow up, prior to RT. **C,** At 5 months follow up after completion of RT, showing residual hyperpigmentation. *RT*, Radiotherapy.

A PET/CT performed 6 weeks later revealed no fluorodeoxyglucose-avid cutaneous lesions, lymphadenopathy, or systemic disease. By 9 weeks, only a hyperpigmented biopsy scar remained; the second papule had spontaneously resolved without intervention (Fig 3). Given the atypical location, absence of systemic involvement, and complete clinical regression, the patient and oncology team elected consolidative local RT to avoid the morbidity associated with systemic chemotherapy. The presence of multiple lesions at presentation, along with reports of relapses following spontaneous regression, supported pursuing definitive local control rather than observation. A total dose of 40 Gy was delivered in 20 fractions of 200 cGy each using an “en face electron” technique. At 5-month follow-up, he remained disease-free with only post-radiation hyperpigmentation (Fig 3). A PET/CT 3 months after completion of RT was negative.

## Discussion

PCDLBCL-LT is an uncommon, aggressive subtype of extranodal B-cell non-Hodgkin lymphoma.^1^, ^2^, ^3^ Dermoscopy may be a useful adjunctive tool but is not diagnostic, as findings are nonspecific; the most commonly described features include white circles, salmon-colored areas, and serpentine vessels.^2^ Definitive diagnosis requires histopathologic confirmation,^1^^,^^2^ which demonstrates an activated B-cell or non-germinal center B-cell-like immunophenotype. Characteristically, there is strong co-expression of CD20, CD79a, BCL-2, multiple myeloma oncogene 1, Forkhead box protein P1, myelocytomatosis, and cytoplasmic IgM.^1^^,^^2^ The proliferation index is high. Variable BCL-6 expression may be seen in some cases; CD10 is typically negative.^1^^,^^2^

Prompt diagnosis, accurate staging, and appropriate risk stratification are essential, as PCDLBCL-LT is associated with poor prognosis, lower complete response rates to therapy, and higher relapse rates.^1^^,^^2^ Non-leg involvement has been linked to improved outcomes, with 1 study noting a 3-year disease-specific survival of 77% for non-leg tumors compared with 43% for leg lesions.^1^^,^^2^ Poor prognostic factors include multiple skin lesions, which confer a significantly lower 3-year disease-specific survival (39%) compared with solitary lesions (77%).^1^^,^^2^ In elderly patients with localized disease, RT alone or in combination with rituximab is often favored to minimize systemic toxicity.^2^ For systemic or disseminated involvement, multiagent regimens such as R-CHOP (cyclophosphamide, doxorubicin, vincristine, prednisone plus rituximab), with or without RT, remain the standard of care.^2^

Spontaneous regression in PCDLBCL-LT is exceptionally rare, with only a handful of cases reported (Table I).^3^, ^4^, ^5^, ^6^, ^7^, ^8^, ^9^, ^10^ Proposed mechanisms include antitumor immune activation, potentially triggered by concomitant viral infections (eg, EBV, rubeola, HIV), biopsy-induced local trauma, or withdrawal of immunosuppression.^3^^,^^9^ CD8+ T-cell–mediated immune responses have been implicated in some reports.^7^^,^^8^^,^^10^ In this patient, it is plausible that the biopsy procedure itself triggered a localized immune response sufficient to induce complete regression of both the biopsied and non-biopsied plaques, suggesting a regional or systemic abscopal-like response.

**Table I:** Summary of cases of primary cutaneous diffuse large B-cell lymphoma, leg-type with non-leg location and spontaneous regression, including this case

Article	Sex/age (y)	Location	Morphology	Immunohistochemistry	Evolution	Repeat biopsy during regression	Complementary therapy	Remission duration
Foo et al^3^ (2014)	F/78	Cheek	Solitary nodule	*Positive*: CD20, BCL (*unspecified*). *Negative*: CD10.	Regressed after withdrawal of methotrexate	NR	Rituximab	NR
Jimura et al^4^ (2017)	F/72	Upper arm	Multiple erythematous nodules	*Positive*: CD20, CD79a, BCL-2, MUM1. *Negative*: CD10.	Regressed after biopsy	Numerous small lymphocytes (positive for CD3, CD4, CD8, perforin, granzyme B, TIA1). Small number of atypical lymphocytes, (positive for CD20)	Surgery	21 mo
Present study (2025)	M/79	Upper back	2 discrete plaques	*Positive*: LCA (CD45), CD20, BCL-2, BCL-6, MUM1, FOXP1, IgM, MYC. *Negative*: IgD, CD5, CD10, CD23, CD30, CD34, CD138, cyclin D1, AE1/AE3 and SOX10	Both the biopsied and the non-biopsied plaques regressed; only 1 lesion was sampled histologically	NR	RT	5 mo

*AE1/AE3*, Cytokeratins; *BCL-2*, B-cell lymphoma 2 protein; *BCL-6*, B-cell lymphoma 2 protein; *FOXP1*, Forkhead box protein P1; *LCA*, leukocyte common antigen; *MUM1*, multiple myeloma oncogene 1; *MYC*, myelocytomatosis; *RT*, radiotherapy; *SOX10*, Sry-box transcription factor 10; *TIA1*, cytotoxic granule-associated RNA binding protein.

A key limitation is the relatively short follow-up of 8 months, which precludes firm conclusions regarding the durability of remission. Nevertheless, these observations may support consideration of less aggressive treatment strategies, such as local therapy alone, in carefully selected patients, particularly those with localized, low-burden disease at non-leg sites, and no systemic involvement, although evidence remains limited and multidisciplinary, case-by-case decision-making is essential. Nevertheless, recurrences have been reported following spontaneous regression,^7^^,^^8^ thus long-term surveillance is critical. Our patient will receive coordinated follow-up with dermatology and oncology, with full skin and lymph node examinations every 3-4 months during the first year. A surveillance chest/abdomen/pelvis CT is scheduled for 6 months after the most recent PET/CT.

While PCDLBCL-LT is generally aggressive, rare cases may demonstrate spontaneous regression. Given the unusual presentation on the upper back and the clinical spontaneous regression as confirmed via PET imaging, recognizing atypical presentations is important for accurate diagnosis, prognostication, and individualizing treatment planning, particularly in elderly populations.

## Conflicts of interest

None disclosed.

## References

[1] Suárez A.L., Pulitzer M., Horwitz S., Moskowitz A., Querfeld C., Myskowski P.L. (2013). Primary cutaneous B-cell lymphomas: part I. clinical features, diagnosis, and classification. J Am Acad Dermatol.

[2] Vitiello P., Sica A., Ronchi A., Caccavale S., Franco R., Argenziano G. (2020). Primary cutaneous B-cell lymphomas: an update. Front Oncol.

[3] Foo S.H., Ladoyanni E., Verpetinske I. Spontaneous regression of atypical primary cutaneous diffuse large B-cell lymphoma on cheek upon methotrexate withdrawal. https://austinpublishinggroup.com/dermatology/fulltext/ajd-v1-id1007.php.

[4] Jimura N., Fujii K., Baba A., Higashi Y., Kanekura T. (2017). Spontaneous regression of a primary cutaneous diffuse large B-cell lymphoma, leg type. J Dermatol.

[5] Winkler M., Albrecht J.D., Sauer C. (2024). Spontaneous regression of primary cutaneous diffuse large B-cell lymphoma, leg type: a case series and review of the literature. J Dermatol.

[6] Li F., Wang L. (2023). Spontaneous regression of primary cutaneous diffuse large B-cell lymphoma, leg type after biopsy. Indian J Dermatol Venereol Leprol.

[7] Graham P.M., Richardson A.S., Schapiro B.L., Saunders M.D., Stewart D.M. (2018). Spontaneous regression of primary cutaneous diffuse large B-cell lymphoma, leg type with significant T-cell immune response. JAAD Case Rep.

[8] Toberer F., Mechtersheimer G., Jaschinski H., Enk A., Hakim-Meibodi L., Haenssle H.A. (2018). Spontaneous regression of primary cutaneous diffuse large B-cell lymphoma, leg type. Acta Derm Venereol.

[9] Ghossein J., Petkiewicz S., Zeng W. (2024). Spontaneous regression of primary cutaneous diffuse large B-cell lymphoma on sequential FDG PET. Am J Dermatopathol.

[10] Marrero-Alemán G., Montenegro-Dámaso T., Peñate Y. (2017). Primary cutaneous diffuse large B-cell lymphoma, leg type, with spontaneous regression after Biopsy. Am J Dermatopathol.

